# Healthy human serum *N*-glycan profiling reveals the influence of ethnic variation on the identified cancer-relevant glycan biomarkers

**DOI:** 10.1371/journal.pone.0209515

**Published:** 2018-12-28

**Authors:** Abrha G. Gebrehiwot, Daniel Seifu Melka, Yimenashu Mamo Kassaye, Ibrahim F. Rehan, Shobith Rangappa, Hiroshi Hinou, Toshiya Kamiyama, Shin-Ichiro Nishimura

**Affiliations:** 1 Division of Drug Discovery Research, Faculty of Advanced Life Science and Graduate School of Life Science, Hokkaido University, Kita-ku, Sapporo, Japan; 2 Department of Biochemistry, School of Medicine, Addis Ababa University, Addis Ababa, Ethiopia; 3 Department of Animal Behaviour and Husbandry, Faculty of Veterinary Medicine, South Valley University, Qena, Egypt; 4 Department of Gastroenterological Surgery I, Graduate School of Medicine, Hokkaido University, N15, W7, Kita-ku, Sapporo, Japan; Edith Cowan University, AUSTRALIA

## Abstract

**Background:**

Most glycomics studies have focused on understanding disease mechanisms and proposing serum markers for various diseases, yet the influence of ethnic variation on the identified glyco-biomarker remains poorly addressed. This study aimed to investigate the inter-ethnic serum *N*-glycan variation among US origin control, Japanese, Indian, and Ethiopian healthy volunteers.

**Methods:**

Human serum from 54 healthy subjects of various ethnicity and 11 Japanese hepatocellular carcinoma (HCC) patients were included in the study. We employed a comprehensive glycoblotting-assisted MALDI-TOF/MS-based quantitative analysis of serum *N*-glycome and fluorescence HPLC-based quantification of sialic acid species. Data representing serum *N*-glycan or sialic acid levels were compared among the ethnic groups using SPSS software.

**Results:**

Total of 51 *N*-glycans released from whole serum glycoproteins could be reproducibly quantified within which 33 glycoforms were detected in all ethnicities. The remaining *N*-glycans were detected weakly but exclusively either in the Ethiopians (13 glycans) or in all the other ethnic groups (5 glycans). Highest abundance (p < 0.001) of high mannose, core-fucosylated, hyperbranched/hypersialylated *N*-glycans was demonstrated in Ethiopians. In contrast, only one glycan (*m/z* 2118) significantly differed among all ethnicities being highest in Indians and lowest in Ethiopians. Glycan abundance trend in Ethiopians was generally close to that of Japanese HCC patients. Glycotyping analysis further revealed ethnic-based disparities mainly in the branched and sialylated structures. Surprisingly, some of the glycoforms greatly elevated in the Ethiopian subjects have been identified as serum biomarkers of various cancers. Sialic acid level was significantly increased primarily in Ethiopians, compared to the other ethnicities.

**Conclusion:**

The study revealed ethnic-specific differences in healthy human serum *N*-glycome with highest abundance of most glycoforms in the Ethiopian ethnicity. The results strongly emphasized the need to consider ethnicity matching for accurate glyco-biomarker identification. Further large-scale study employing various ethnic compositions is needed to verify the current result.

## Introduction

Glycosylation, the process in which sugars are attached to proteins or lipids, is the most abundant and complex post-translational process, causing immense structural and functional variabilities in majority of eukaryotic cell proteins [1, 2]. Glycan parts of glycoconjugates are known to facilitate essential roles in almost all physio-pathological processes including fertilization, cell differentiation, cell adhesion, cell recognition, molecular trafficking, signal transduction, protein folding, immunological regulation, aging, and even malignant alterations [1–3]. The indispensable role of glycosylation in complex organisms like humans is apparent from the fact that many eukaryotic cells can function and survive without nucleus; however, none of them can function normally without glycans at least on their surface [4–6], and hence total absence of glycans is embryologically lethal [7]. Many of the human serum proteins including alpha-1-acid glycoprotein, alpha-1-antitrypsin, alpha-2-macroglobulin, antithrombin-III, apolipoproteins, ceruloplasmin, fibrinogen, immunoglobulins, haptoglobin, hemopexin, and serotransferrin, are heavily glycosylated, making them targets for glyco-biomarker discovery and therapeutic opportunities [8].

In contrast to nucleic acids and proteins, biosynthesis of glycans is not template-driven but, rather, is a result of a complex network of metabolic and enzymatic reactions. Because of this and subsequent methodological difficulties, the field of glycomics has been lagging behind genomics and proteomics [9, 10]. Tremendous advancements in analytical techniques and bioinformatics platforms have recently revolutionized the area, enabling comprehensive profiling of glycans and glycoproteins to be released from various biological samples [11–13] and suggested as biomarkers. However, bringing these glycan or glycoprotein markers to clinical practice has been hindered as their potential to distinguish cases from controls or disease stages seems to be inadequate and varies from country to country population wise, complicating their validity and clinical utility [14, 15]. For example, the FDA approved cancer biomarkers based on serum level of *O*-glycosylated mucin glycoproteins of carbohydrate antigens (CA125 for ovarian, CA27.29 and CA15-3 for breast, CA19-9 for pancreatic cancers) and *N*-glycosylated glycoproteins (α-fetoprotein for hepatocellular carcinoma, prostate-specific antigen for prostate cancer) lack the specificity and sensitivity to be used for early detection of cancer [16, 17].

Previous population-based studies have reported the association of plasma *N*-glycan structure alterations (mostly increased plasma *N*-glycan complexity) with metabolic syndrome and higher risk of type 2 diabetes [18, 19]. In several glycomics studies profiling immunoglobulin G (IgG) focused *N*-glycome among various ethnic populations, it was emphasized that changes in IgG-linked glycan composition and abundance were correlated with hypertension [20–22], cardiovascular disease [23], blood lipids and dyslipidaemia [24]. Apart from several pathological studies that have profiled glycosylation pattern, comparative glycosylation studies among healthy subjects with various characteristics are often overlooked. Nevertheless, such studies on healthy people would be of clinical benefit not only by providing anticipatory insights but also by elucidating confounding factors that could lead to controversies on the identified biomarker [25]. In this context, a comprehensive study on human plasma *N*-glycan profile provided an evidence for the variability of some glycan levels with aging, life style and environmental factors [26]. Similarly, Ding N. and his group demonstrated that a healthy human serum *N*-glycan profile had shown considerable variations in age and sex dependent manner [27]. Furthermore, IgG focused *N*-glycomic study in a Han Chinese population revealed that changes in IgG *N*-glycan features significantly correlate with age [28]. To our knowledge, little is known about the association between ethnicity and healthy human serum *N*-glycome profile. Thus, this study was aimed to address the inter-ethnic differences in serum *N*-glycome among US origin control, South Indian, Japanese, and Ethiopian ethnic populations using a rapid glycoblotting-assisted MALDI-TOF/MS-based quantitative analysis. The present study clearly demonstrated that various high mannose, core-fucosylated, multiply branched and sialylated glycoforms illustrated an ethnic-specific expression pattern and marked alterations in their serum abundance.

## Materials and methods

### 2.1 Human serum samples

The study was performed in accordance with the ethical guidelines and protocols of the Declaration of Helsinki upon approval by the ethical review boards of Hokkaido University, Faculty of Advanced Life Sciences, Japan and Addis Ababa University, School of Medicine, Ethiopia. Informed consent was obtained from all volunteer participants. As summarized in S1 Table, total of 54 healthy subjects having a relatively narrow age range and various ethnic composition were involved in the study. Serum from male Japanese (n = 10, age = 20s-30s y/o), and male South Indians (n = 10, age = 32.5 ± 5 y/o) were collected as part of Asia-Africa Science Platforms project. Similarly, serum samples from 24 female Ethiopians (31.54 ± 7 y/o) were collected in Black Lion Specialized Teaching Hospital of Addis Ababa University and carefully transported to Japan after freeze dried. US origin healthy control samples were from serum pool of different male donors whose age information was not provided as these samples were purchased from Sigma-Aldrich company (product # H4522). Serum samples from male Japanese hepatocellular carcinoma (HCC) patients (n = 11, age = 50s-60s y/o) were also included in the study. As inclusion criteria for the healthy ethnic groups, fully heathy (mentally and physically) adults (> 18 y/o), non-smokers, and non-obese individuals were included in the present study. Whereas, individuals who were pregnant, were receiving any treatment, or were with a medical history of disease such as cancer, diabetes, neurodegenerative, liver, or cardiovascular diseases which can affect the glycan profile, were excluded from the study. All serum samples were kept at -80°C until used in the subsequent experiment.

### 2.2 Reagents and Equipment

Ammonium bicarbonate 99% (ABC), 1-propanesulfonic acid, 2-hydroxyl-3-myristamido (PHM), 3-methyl-1-*p*-tolyltriazene (MTT), *O*-benzylhydroxylamine hydrochloride (BOA), and disialyloctasaccharide were purchased from Tokyo Chemical Industry Co., Ltd. (Tokyo, Japan). Peptide *N*-glycosidase F (PNGase F) was purchased from New England Biolabs Inc. (Ipswich, Massachusetts, USA), whereas BlotGlyco H beads were purchased from Sumitomo Bakelite, Co. Ltd. (Tokyo, Japan). Dithiothreitol (DTT), iodoacetamide (IAA), trypsin, α-cyano-4-hydroxycin-namic acid diethylamine salt, 2-mercaptoethanol, and *5-N-*Glycolylneuraminic acid (Neu5Gc) were from Sigma-Aldrich, Inc. (St. Louis, MO, USA). *5-N-*acetylneuraminic acid (Neu5Ac) was purchased from Japan Food & Liquor Alliance Inc., Food & Bio Research Center, Inc. (Kyoto, Japan). 1,2-Diamino-4,5-methylenedioxybenzene (DMB) was from Dojindo Laboratories (Kumamoto, Japan). Other reagents and solvents were obtained from Wako Pure Chemicals Co., (Tokyo, Japan), unless otherwise stated. SweetBlot^TM^ (automated glycan processing and incubating machine) was from System Instruments Co., Ltd., (Hachioji, Japan). MultiScreen Slvinert^R^ filter plates were purchased from Millipore Co., Inc. (Tokyo, Japan). All mass quantifications were done using MALDI-TOF/MS (Ultraflex III, Brukers Daltonics, Germany). HPLC analysis was performed on Hitachi D-7000, Hitachi High Technologies Co., Ltd. (Tokyo, Japan).

### 2.3 Release of total *N*-glycans from human serum glycoproteins

A method based on high-throughput glyco-technology previously developed and optimized in our laboratory [11–13, 29–32] was used for glycan release, purification, labeling and spotting. Initially, 10 μL of each serum sample was transferred to a 96 well polymerase chain reaction (PCR) plate and dissolved with 30 μL of freshly prepared 0.33M ammonium bicarbonate (ABC) containing 0.1% of PHM in 10mM ABC and the solution was incubated at 37°C for 10 min., serially diluted human serum standards were prepared by mixing about 50 μL serum aliquot of each healthy participant to give pooled sera, from which the concentration of standard serial dilutions (0.5×, 0.75×, 1.0×, 1.25×, 1.5×, 1.75×, 2.0×, and 2.25×) were adjusted using Milli-Q water and then included in the experiment as the resulting calibration curve helps to evaluate the linearity and reproducibility of the detect peaks. As an internal standard, 12 μL of 60 μM disialyloctasaccharide was also added and mixed in each well to aid eventual quantification of detection *N*-glycans. Solubilized proteins were reduced by 10 μL of 120 mM 1, 4-dithiothreitol (DTT) at 60°C for 30 min followed by alkylation with 20 μL of 123 mM iodoacetamide by incubation in dark for 1 hr. The mixture was then treated with 10 μL of 40 U/μL trypsin in 1 mM HCl at 37°C for 2 hrs. After heat-inactivation of the enzyme at 90°C for 10 minutes and then cooling to room temperature, *N*-glycans were released from trypsin-digested samples by incubation with 2 U of Peptide *N*-glycosidase F (PNGase F) at 37°C for 6 hrs.

### 2.4 Selective *N*-glycan enrichment by glycoblotting method

**Fig 1:**
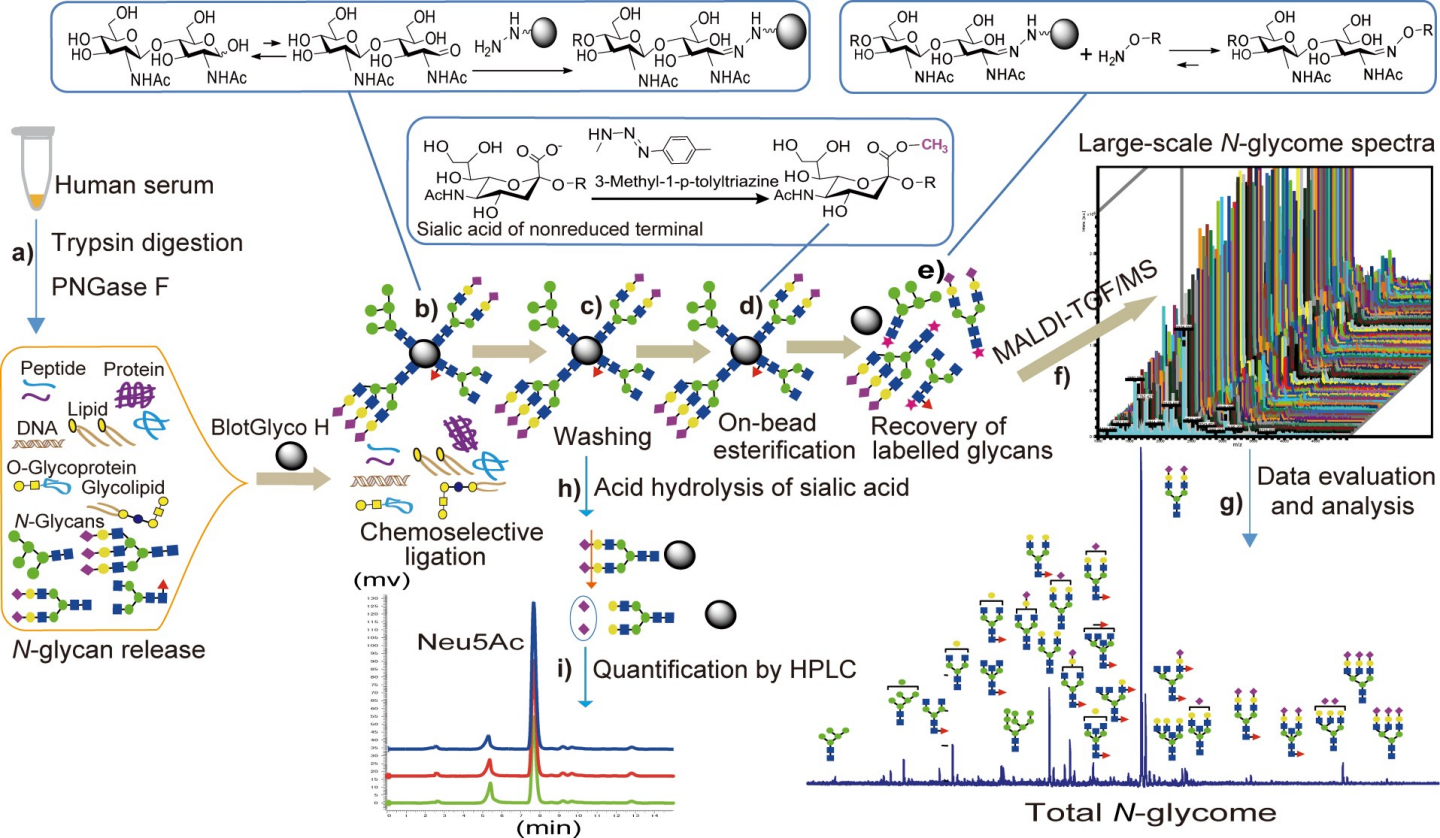
Schematic workflow of glycoblotting-assisted MALDI-TOF/MS and HPLC analysis. (**a**) Enzymatic treatment for serum protein digest and deglycosylation using trypsin and PNGase F, respectively, (**b**) Chemoselective capturing of reducing sugars onto a hydrazide-functionalized BlotGlyco H beads from complex mixture of biomolecules, (**c**) Washing to remove all impurities, (**d**) On-bead methyl esterification of sialic acid residues, (**e**) Recovery of BOA-labeled glycans, (**f**) MALDI-TOF/MS analysis, (**g**) Data processing and evaluation, (**h**) Cleavage of free sialic acid residues from captured *N*-glycans, (**i**) Free sialic acid labeling and quantification by fluorescence-HPLC.

Once *N*-glycans were enzymatically released, oligosaccharides carrying reducing terminal were chemically ligated with hydrazide-functionalized BlotGlyco H bead, allowing their selective capturing from the complex mixtures of serum originated biomolecules as diagrammed in Fig 1. In this glycoblotting-based quantitative *N*-glycomics strategy, 250 μL of BlotGlyco H bead was placed into each well of a MultiScreen Solvinert filter plate (Millipore) with vacuuming. 20 μL of PNGase F digested mixture containing released *N*-glycans was then mixed with the bead in each well, followed by the addition of 180 μL of 2% acetic acid (AcOH) in acetonitrile (ACN). To capture the *N*-glycans specifically onto beads via reversible hydrazone bonds, the plate was incubated at 80°C for 45 minutes. Next, the plate was washed twice with each 200 μL of 2 M guanidine-HCl in ABC, water, and 1% triethyl amine in methanol (TEA in MeOH). To cap unreacted hydrazide functional groups on beads, 10% acetic anhydride in MeOH was added with incubation for 30 min at room temperature and then removing the solution by vacuum. The beads were then washed twice with each 200 μL of 10 mM HCl, methanol, and dioxane. To prevent sialic acid dissociation under mild acidic condition or when directly ionized by MALDI-TOF/MS, on-bead esterification of its carboxyl groups was carried out by incubation with fresh 100 mM 3-methyl-1-*p*-tolyltriazene (MTT) in dioxane at 60°C for 90 minutes. This approach allows simultaneous analysis of neutral and acidic (sialylated) glycans in positive-ion detection mode. Subsequently, each well was washed twice using 200 μL of dioxane, water, methanol, and water. The glycans blotted on the beads were labeled by trans-iminization reaction with 20 μL of 50 mM *O*-benzyloxyamine hydrochloride (BOA) and 180 μL of 2% AcOH in ACN with incubation at 80°C for 45 minutes. BOA-tagged *N*-glycans were finally eluted with 100 μL of Milli-Q water.

### 2.5 Serum *N*-glycan analysis by MALDI-TOF/MS

BOA-labeled *N*-glycans were directly dissolved with an equivalent volume of matrix solution (100 mM α-cyano-4-hydroxycin-namic acid diethylamine salt), after which 2.5 μL of each sample-matrix mixture was auto-spotted in quadruplicate on MTP 384 target plate (polished steel TF, Bruker Daltonics). Ultraflex III mass spectrometry that works based on matrix-assisted laser desorption/ionization-time of flight/mass spectrometry (MALDI-TOF/MS) was used during which mass spectra were acquired in an automated manner using AutoXecute flexControl software (Bruker Daltonics, Germany) in reflector, positive ion mode, typically summing 1000 shots for each spot. The obtained mass spectra were further analyzed using FlexAnalysis v. 3 Software (Bruker Daltonics, Germany). The intensities from monoisotopic peaks of each quadruplicated spectra were normalized using known concentration of an internal standard and then averaged. This data was used for further statistical and quantitative comparison. Detected *N*-glycans were selected based on their quantitative reproducibility after evaluated using calibration curve of serially diluted human serum standards. *N*-glycan structural compositions were assigned by GlycoMod (ExPASy proteomics server, Swiss Institute of Bioinformatics: http://br.expasy.org/tools/glycomod/) using experimental masses, and by CFG database (http://www.functionalglycomics.org/glycomics/publicdata/home.jsp).

### 2.6 Quantification of free sialic acids cleaved from *N-*glycans of human serum

**Fig 2:**
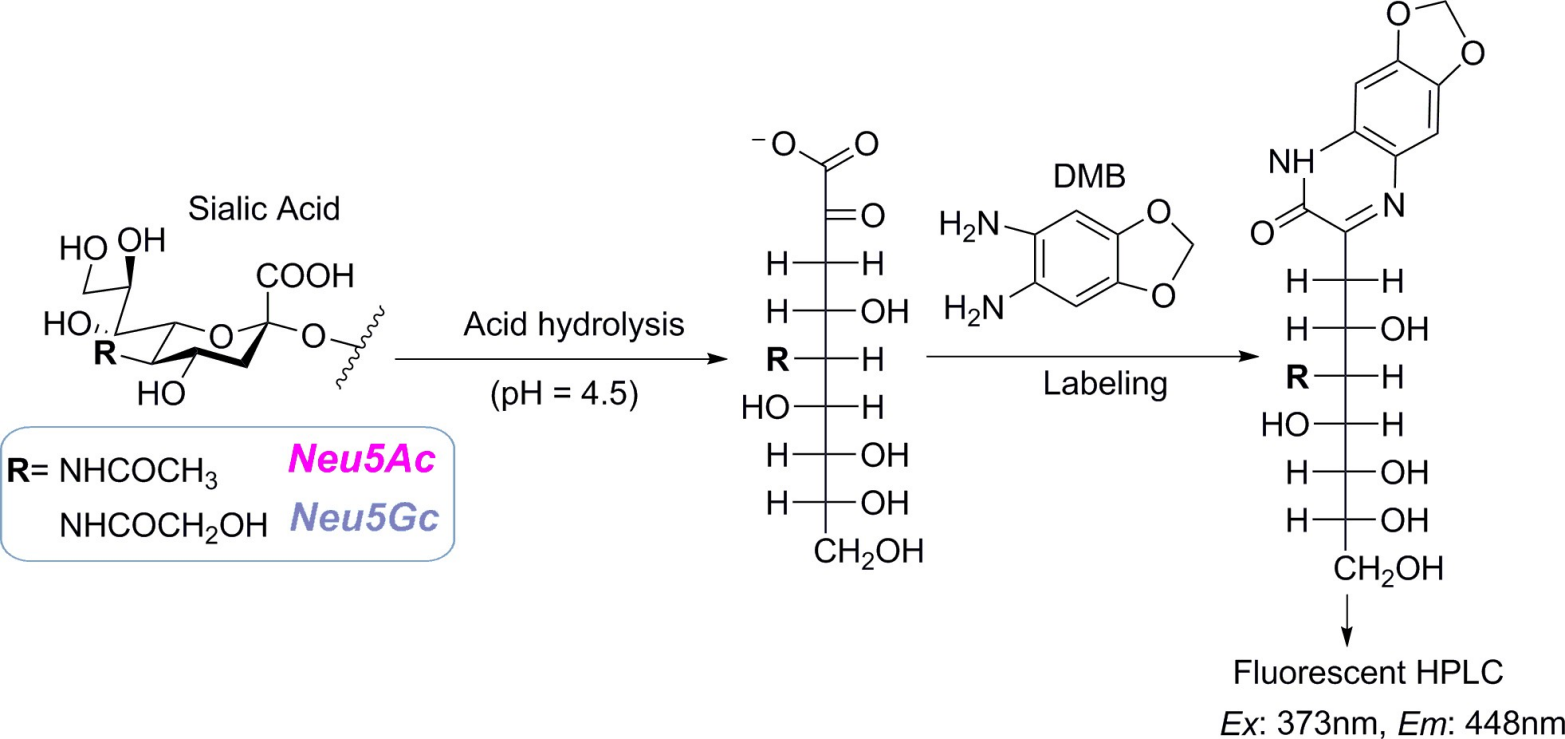
Scheme for hydrolytic cleavage of terminal sialic acid and its labeling for fluorescence detection. **DMB**: 1,2-diamino-4,5-methylenedioxybenzene, **Neu5Ac**: *N-*acetylneuraminic acid, **Neu5Gc**: *N-*glycolylneuraminic acid.

To demonstrate the versatility of our comprehensive glycoblotting method for HPLC based sialic acid quantification, previously reported methods [33, 34] were slightly modified and integrated to our automated glycan enrichment approach. Briefly, after enzymatic release of serum *N*-glycans under the digestion conditions described above, 20 μL from each sample containing released *N-*glycans was subjected to glycoblotting with BlotGlyco H beads in a similar procedure as described above except sialic acid esterification with MTT and subsequent procedures were excluded in this case. Next to acetyl capping and subsequent washing, 100 μL of 25 mM HCl was added to each well, with sealing and incubating of the plate at 80°C for 1 hr to release terminal sialic acids via selective cleavage of the α-glycosidic bond from the adjacent galactose residues (Fig 1H). The hydrolysate containing trimmed sialic acid was filtered and collected into PCR tubes. The filtrate was then reacted with 100 μL of 7 mM 1,2-diamino-4,5-methylenedioxybenzene (DMB) reagent (prepared by dissolving DMB−2HCl powder in equal volumes of 1 M 2-mercaptoethanol and 18 mM Na_2_S_2_O_4_) with heating at 60°C for 2.5 hrs in dark, allowing labeling of sialic acids. This DMB reagent effectively derivatizes all type of sialic acids without any side reaction as it specifically reacts with their α-keto acid moiety (see Fig 2). After stopping the reaction by cooling in ice water, 50 μL of the mixture solution was transferred into vial tube from which 10 μL was auto-injected into fluorescence HPLC for analysis on a reversed-phase column. The column was eluted at a flow rate of 1 mL/min using MeOH/ACN/H_2_O solution mixture (3:1:10, v/v). The fluorescence was detected at 448 nm using excitation at 373 nm in a D-7000 HPLC system equipped with an L-7485 fluorescence detector (Hitachi High-Technologies Co., Tokyo, Japan). Standard mixtures of *N-*acetylneuraminic acid (Neu5Ac) and *N-*glycolylneuraminic acid (Neu5Gc, a hydroxylated form of the common sialic acid, Neu5Ac) were run parallel with the samples to normalize the retention time of each peak on the column, allowing quantitative analysis.

### 2.7 Statistical analysis

*N*-glycan peaks detected in MALDI-TOF/MS spectra were annotated using FlexAnalysis 3.0 software (Bruker Daltonics, Germany). Normalized data for expression levels of *N*-glycans were analyzed using SPSS software. Multiple comparisons among the ethnic groups were done using one-way analysis of variance (ANOVA). P-values were adjusted for multiple testing using Bonferroni method and mean value differences were considered significant at 95% confidence interval (*p* ≤ 0.05). We also used Graph Pad prism 5 to show the serum *N*-glycan and free sialic acid level of individual samples in a scatter dot plot.

## Results

### 3.1 Quantitative reproducibility test using standard human serum

**Fig 3:**
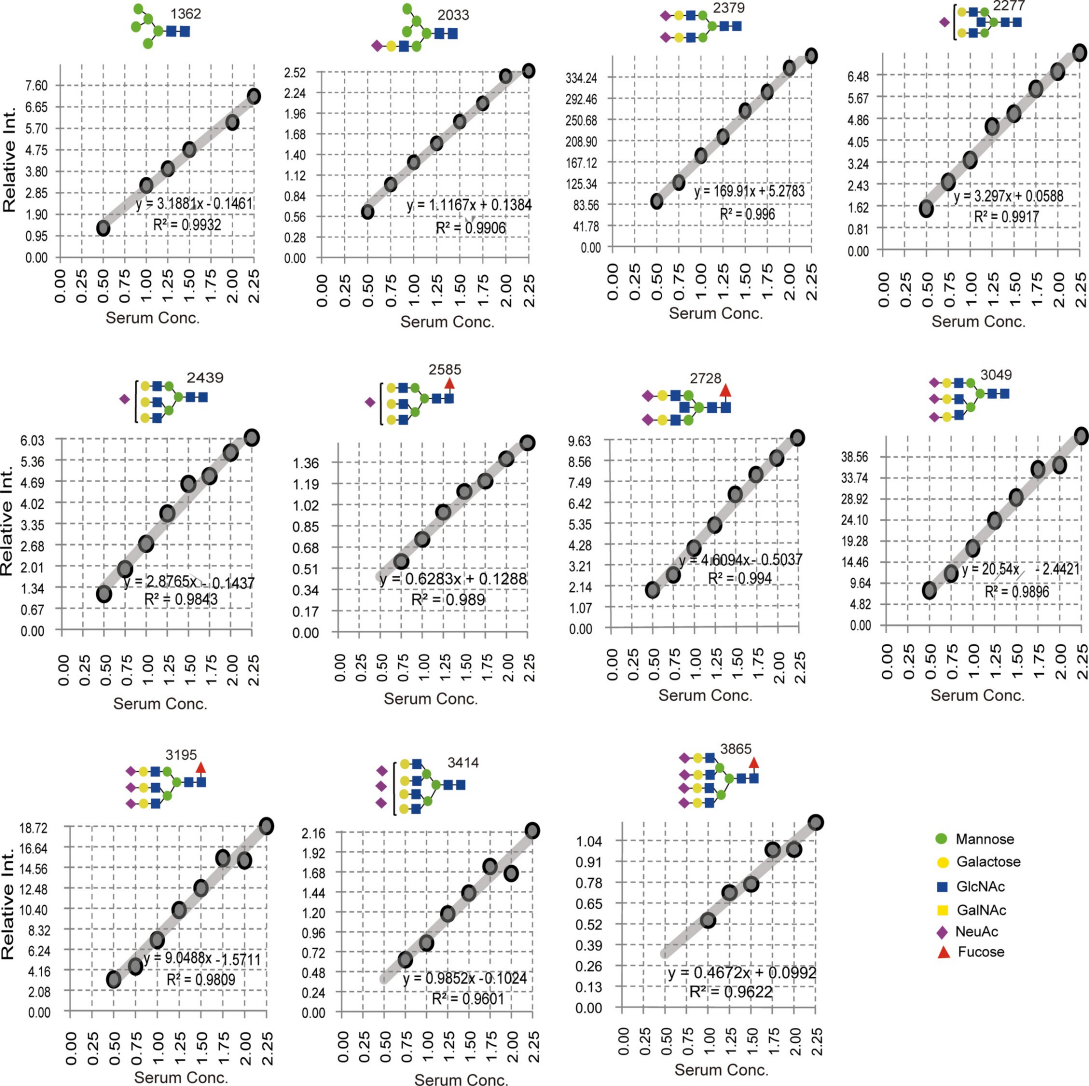
Human serum standard calibration curve of some common *N*-glycans. Glycans that have shown quantitatively reproducible detection across the various standard serum concentrations were considered and selected in the case of the main study samples. For identification purpose, individual glycans are coded or labeled by their molecular weight (*m/z* value) shown near to their structure.

To select the reliably detected *N*-glycans, each peak was evaluated for its quantitative reproducibility using serially diluted standard human serum samples (0.5×, 0.75×, 1.0×, 1.25×, 1.5×, 1.75×, 2.0×, and 2.25×) that were simultaneously experimented in the same plate beside the study samples. The peak intensity of each glycan was first normalized using known concentration of an internal standard. Standard calibration curve for each *N*-glycan across the dilution series was plotted using the normalized data as shown in Fig 3. *N*-glycans peaks that met the criteria of at least p<0.05 detection reliability, accessibility in at least six of the total eight human serum standards, and minimum outlier scores were selected and considered for quantitative comparison in the result of main study samples.

### 3.2 Inter-ethnic variation in the total serum *N*-glycan profile

**Fig 4:**
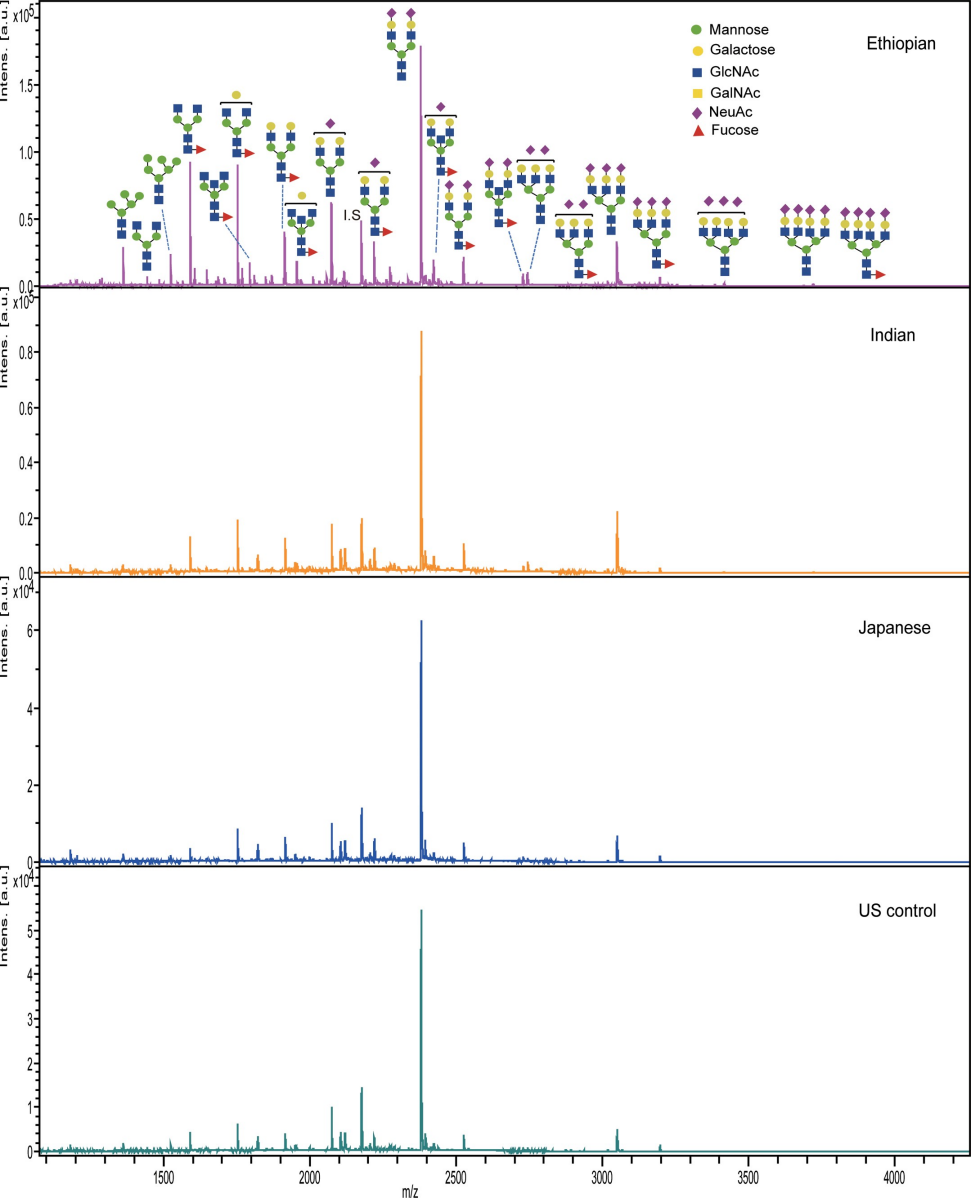
Representative MALDI-TOF/MS spectra of human serum *N*-glycans acquired from subjects of various ethnicity. Structures for some *N*-glycans that demonstrated differential peak intensities are indicated over the respective glycan peak.

The comprehensive *N*-glycomics study mainly emphasized investigating the association between serum glycan expression pattern and ethnic variation among 54 healthy individuals comprising Japanese, South Indians, Ethiopians, and US origin controls. Mass spectral data acquired by MALDI-TOF/MS analysis demonstrated noticeable variation in the peak intensities of several glycans with the highest signals appeared in the Ethiopian ethnic group (Fig 4). Taking the entire study samples, detection of 51 *N*-glycans (Table 1) was quantitatively verified of which 33 *N*-glycans were common to all ethnic groups. Detection level of the remaining glycans was < 5 μM and exclusive either to the Ethiopian ethnic group (13 glycans, Table 1 highlighted in gold color shade) or to all the other ethnic groups (5 glycans, Table 1 highlighted in light green color shade). Here, it should be clear that glycans whose detection profile was not quantitatively reliable or limited to some samples were not considered to avoid biased conclusions. Overall, 40 (78.43%) of the detected *N*-glycans belong to complex type whereas high-mannose and hybrid types comprise 5 (9.8%) and 6 (11.76%), respectively.

**Table 1:** Estimated composition of 51 *N*-linked glycans identified from human serum glycoproteins.

Peak #	m/z	Glycan composition	Type
1	1362.48109	(Hex)2 + (Man)3 (GlcNAc)2	High-Mannose
2	1444.53419	(HexNAc)2 + (Man)3 (GlcNAc)2	Complex
3	1524.53392	(Hex)3 + (Man)3 (GlcNAc)2	High-Mannose
4	1565.56047	(Hex)2 (HexNAc)1 + (Man)3 (GlcNAc)2	Hybrid
5	1590.59210	(HexNAc)2 (Deoxyhexose)1 + (Man)3 (GlcNAc)2	Complex
6	1606.58702	(Hex)1 (HexNAc)2 + (Man)3 (GlcNAc)2	Complex
7	1686.58675	(Hex)4 + (Man)3 (GlcNAc)2	High-Mannose
8	1708.61871	(Hex)1 (HexNAc)1 (NeuAc)1 + (Man)3 (GlcNAc)2	Complex
9	1752.64493	(Hex)1 (HexNAc)2 (Deoxyhexose)1 + (Man)3 (GlcNAc)2	Complex
10	1768.63985	(Hex)2 (HexNAc)2 + (Man)3 (GlcNAc)2	Complex
11	1793.67148	(HexNAc)3 (Deoxyhexose)1 + (Man)3 (GlcNAc)2	Complex
12	1848.63958	(Hex)5 + (Man)3 (GlcNAc)2	High-Mannose
13	1854.67662	(Hex)1 (HexNAc)1 (Deoxyhexose)1 (NeuAc)1 + (Man)3 (GlcNAc)2	Complex
14	1870.67154	(Hex)2 (HexNAc)1 (NeuAc)1 + (Man)3 (GlcNAc)2	Hybrid
15	1873.874	(Hex)3 (HexNAc)1 (Deoxyhexose)1 + (Man)3 (GlcNAc)2	Hybrid
16	1911.69809	(Hex)1 (HexNAc)2 (NeuAc)1 + (Man)3 (GlcNAc)2	Complex
17	1914.69776	(Hex)2 (HexNAc)2 (Deoxyhexose)1 + (Man)3 (GlcNAc)2	Complex
18	1955.72431	(Hex)1 (HexNAc)3 (Deoxyhexose)1 + (Man)3 (GlcNAc)2	Complex
19	1996.967	(HexNAc)4 (Deoxyhexose)1 + (Man)3 (GlcNAc)2	Complex
20	2010.69241	(Hex)6 + (Man)3 (GlcNAc)2	High-Mannose
21	2032.72437	(Hex)3 (HexNAc)1 (NeuAc)1 + (Man)3 (GlcNAc)2	Hybrid
22	2057.75600	(Hex)1 (HexNAc)2 (Deoxyhexose)1 (NeuAc)1 + (Man)3 (GlcNAc)2	Complex
23	2073.75092	(Hex)2 (HexNAc)2 (NeuAc)1 + (Man)3 (GlcNAc)2	Complex
24	2117.77714	(Hex)2 (HexNAc)3 (Deoxyhexose)1 + (Man)3 (GlcNAc)2	Complex
25	2166.284	(Hex)3 (HexNAc)1 (Deoxyhexose)3 + (Man)3(GlcNAc)2	Hybrid
26	2175.78261	(Hex)2 (HexNAc)2 (NeuAc)2 + (Man)3 (GlcNAc)1	I.S
27	2219.80883	(Hex)2 (HexNAc)2 (Deoxyhexose)1 (NeuAc)1 + (Man)3 (GlcNAc)2	Complex
28	2260.83538	(Hex)1 (HexNAc)3 (Deoxyhexose)1 (NeuAc)1 + (Man)3 (GlcNAc)2	Complex
29	2263.83505	(Hex)2 (HexNAc)3 (Deoxyhexose)2 + (Man)3 (GlcNAc)2	Complex
30	2276.83030	(Hex)2 (HexNAc)3 (NeuAc)1 + (Man)3 (GlcNAc)2	Complex
31	2301.444	(HexNAc)4 (Deoxyhexose)1 (NeuAc)1 + (Man)3 (GlcNAc)2	Complex
32	2336.85144	(Hex)3 (HexNAc)4 + (Man)3 (GlcNAc)2	Complex
33	2378.86199	(Hex)2 (HexNAc)2 (NeuAc)2 + (Man)3 (GlcNAc)2	Complex
34	2422.88821	(Hex)2 (HexNAc)3 (Deoxyhexose)1 (NeuAc)1 + (Man)3 (GlcNAc)2	Complex
35	2438.88313	(Hex)3 (HexNAc)3 (NeuAc)1 + (Man)3 (GlcNAc)2	Complex
36	2483.89335	(Hex)3 (HexNAc)1 (Deoxyhexose)1 (NeuAc)2 + (Man)3 (GlcNAc)2	Hybrid
37	2520.93623	(Hex)1 (HexNAc)5 (NeuAc)1 + (Man)3 (GlcNAc)2	Complex
38	2524.91990	(Hex)2 (HexNAc)2 (Deoxyhexose)1 (NeuAc)2 + (Man)3 (GlcNAc)2	Complex
39	2581.94137	(Hex)2 (HexNAc)3 (NeuAc)2 + (Man)3 (GlcNAc)2	Complex
40	2584.94104	(Hex)3 (HexNAc)3 (Deoxyhexose)1 (NeuAc)1 + (Man)3 (GlcNAc)2	Complex
41	2727.99928	(Hex)2 (HexNAc)3 (Deoxyhexose)1 (NeuAc)2 + (Man)3 (GlcNAc)2	Complex
42	2743.99420	(Hex)3 (HexNAc)3 (NeuAc)2 + (Man)3 (GlcNAc)2	Complex
43	2787.89	(Hex)3 (HexNAc)4 (Deoxyhexose)1 (NeuAc)1 + (Man)3 (GlcNAc)2	Complex
44	2890.05211	(Hex)3 (HexNAc)3 (Deoxyhexose)1 (NeuAc)2 + (Man)3 (GlcNAc)2	Complex
45	3007.09472	(Hex)4 (HexNAc)5 (NeuAc)1 + (Man)3 (GlcNAc)2	Complex
46	3049.10527	(Hex)3 (HexNAc)3 (NeuAc)3 + (Man)3 (GlcNAc)2	Complex
47	3109.12641	(Hex)4 (HexNAc)4 (NeuAc)2 + (Man)3 (GlcNAc)2	Complex
48	3195.16318	(Hex)3 (HexNAc)3 (Deoxyhexose)1 (NeuAc)3 + (Man)3 (GlcNAc)2	Complex
49	3414.23748	(Hex)4 (HexNAc)4 (NeuAc)3 + (Man)3 (GlcNAc)2	Complex
50	3560.29539	(Hex)4 (HexNAc)4 (Deoxyhexose)1 (NeuAc)3 + (Man)3 (GlcNAc)2	Complex
51	3719.34855	(Hex)4 (HexNAc)4 (NeuAc)4 + (Man)3 (GlcNAc)2	Complex
52	3865.40646	(Hex)4 (HexNAc)4 (Deoxyhexose)1 (NeuAc)4 + (Man)3 (GlcNAc)2	Complex

The *m/z* values indicate experimental masses of *N*-glycans tagged with BOA for enhancing their ionization potentials. Glycans whose peaks # is highlighted by color shade had generally weaker detection profile in which those highlighted in gold color shade were specific to the Ethiopian group while those highlighted in light green shade were common to the remaining groups. Peak number 26 detected at *m/z* 2175.78 represents an internal standard (I.S).

**Fig 5:**
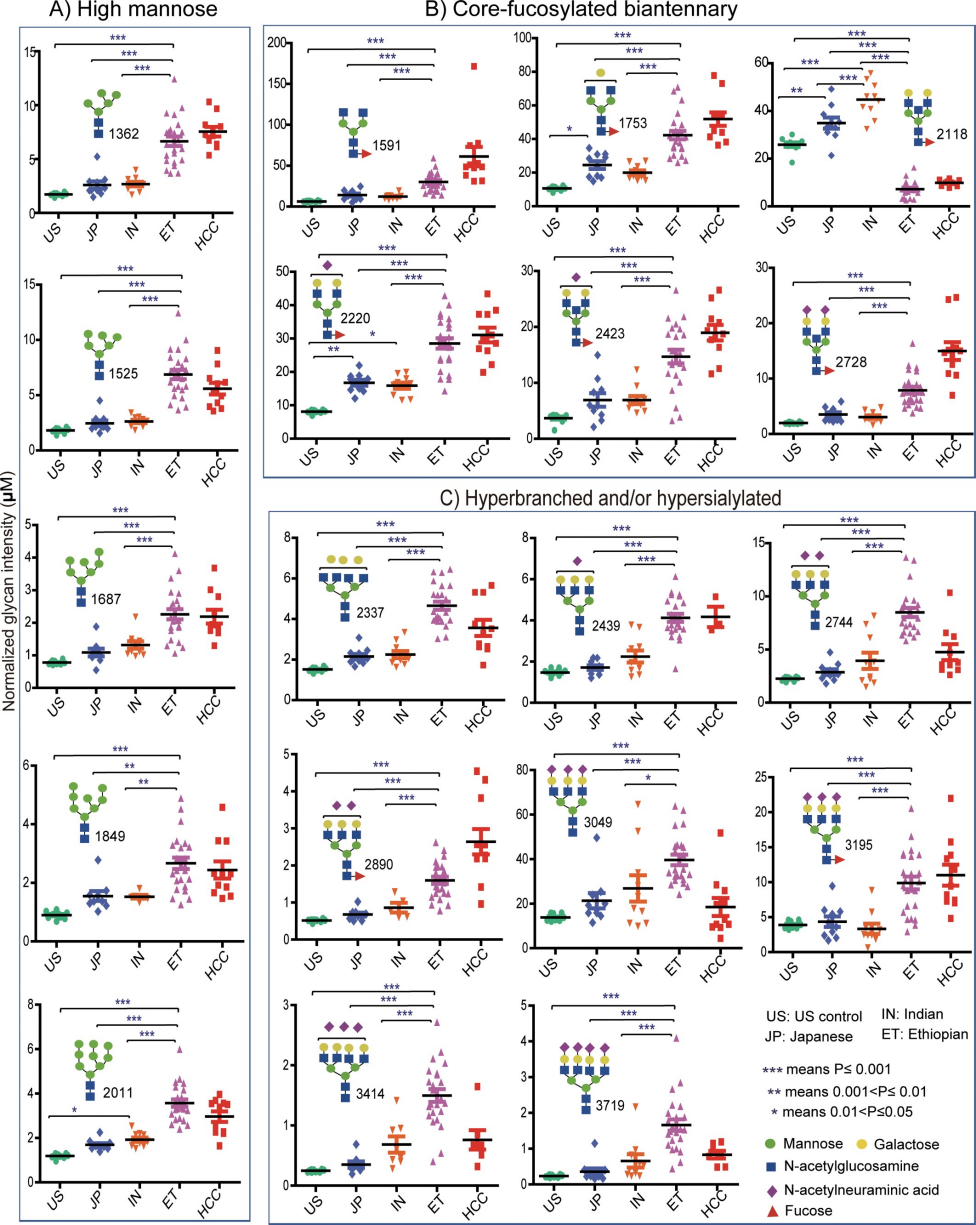
Dot plot illustrating comparative expression abundance of selected serum *N*-glycans. Proposed glycan structure and statistically significant differences among the groups is indicated for each glycoform. **A**) High mannose type, **B**) Core-fucosylated and biantennary structures, **C**) Hyperbranched and/or hypersialylated structures. **HCC**: Hepatocellular carcinoma patients.

We quantitatively compared the expression levels of commonly detected *N*-glycans among the ethnic groups by ANOVA test. Ethnicity-associated variation over a wide range of serum *N*-glycans with a consistent highest abundance (except one glycan drastically declined; *m/z* of 2118) in the Ethiopian subjects (*p* < 0.001) was observed [Fig 5A–5C]. Structurally, these significantly altered glycoforms are predominantly composed of high mannose, core-fucosylated, hyperbranched or hypersialylated types. Moreover, the trend for glycan abundance in Ethiopians tends to be isolated from the other healthy ethnic groups, rather seems closer to that of Japanese hepatocellular carcinoma (HCC) patients [29] whose serum was concurrently re-examined for *N*-glycan profiling. Compared to the US controls, the Japanese and the Indian healthy human serum glycome showed a unidirectional increase in expression level whereas a statistically non-significant expression difference was noticed between the two Asian ethnic groups. As an exception, the result pointed out that a bisect-type *N*-glycan having *m/z* of 2118 showed a significant (*p* < 0.001) concentration alteration among all ethnic groups with the highest in the Indian and the lowest in the Ethiopian groups [see *m/z* 2118 in Fig 5B].

Since Ethiopian subjects were females, for more clarity on whether differences in ethnicity, gender, or age seem to have more marked effects, we further included one Indian female (age = 43) and one Japanese female (age = 40s), whose serum *N*-glycome pattern in comparison with one Ethiopian female (age = 40) is diagramed in S1 Fig. For further emphasis on the gender effect, spectral *N*-glycome profile of Indian and Japanese subjects of both sexes is provided in S2 Fig. within which peak intensity of many *N*-glycans varied mainly in an ethnicity dependent manner, but irrespective of the gender difference. Altogether, these results show clear differences in the glycan abundance that were more strongly associated with ethnic differences than gender or age variation. The effect of age on serum glycan level within each ethnic group of this study was statistically non-significant.

**Fig 6:**
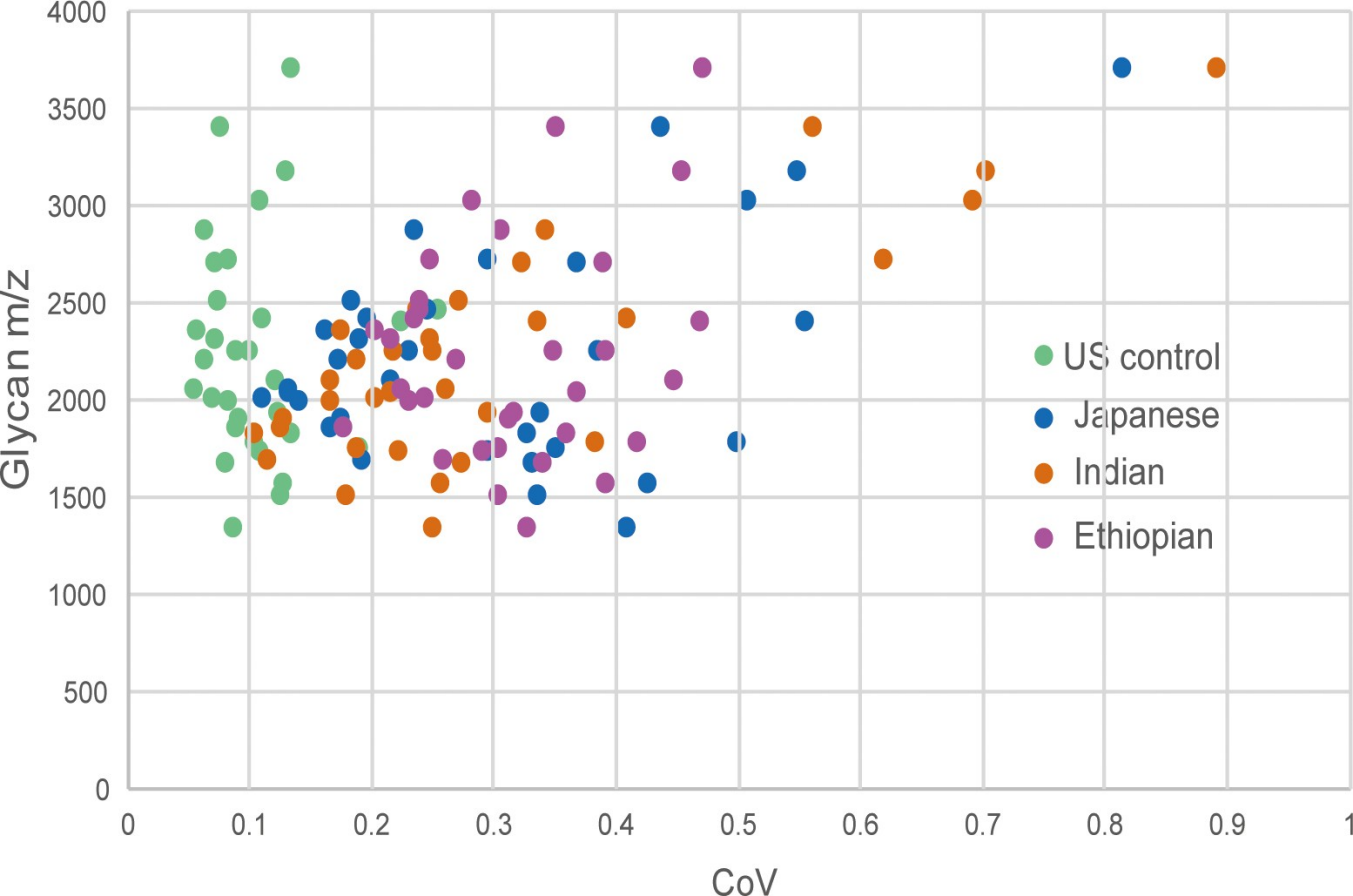
CoV illustrating the inter-subject extent of variation within each ethnic group. Y-axis shows *m/z* values of detected *N*-glycans, X-axis shows the coefficient of variation (CoV) value representing how far the glycan concentration was scattered within subjects of each ethnic group.

To address the inter-individual variation of each *N*-glycan level from one ethnic data series to another, we determined coefficient of variation (CoV) for each glycan as the ratio of the standard deviation to the mean of glycan expression. The degree of variation was found to be lowest across the entire glycans in the US control sample. On the other hand, there were no consistent inter-subject differences among Japanese, Indian and Ethiopian ethnicities up to *m/z* of 2500, after which the hyperbranched and hypersialylated glycans showed widely dispersed expression pattern in the Indian and the Japanese groups (Fig 6).

### 3.3 Glycotyping analysis

**Fig 7:**
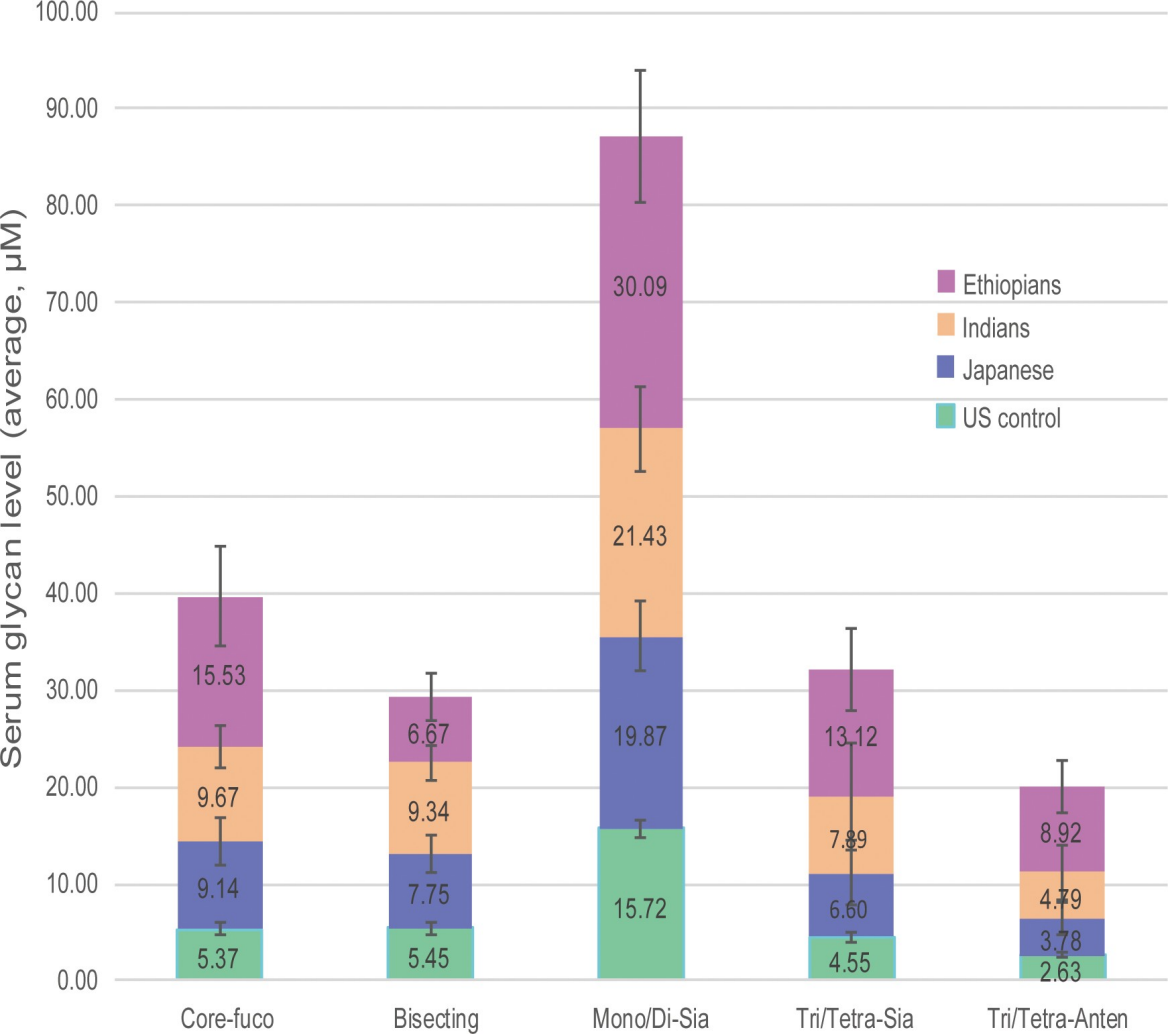
Ethnic-based serum level of some common glyco-subclasses. *N*-glycans were grouped in terms of core-fucosylation, bisecting, mono- or di-sialylation, tri-or tetra-sialylation, tri- or tetra-antennary features. Error bars indicate standard errors of the means.

After *N*-glycans were stratified into glyco-subclasses that share certain structural features of core-fucosylation, bisecting, sialylation, and branching (more details provided in S2 Table), their overall expression pattern among the study groups is shown in Fig 7. The result clearly revealed that profound abundance of these glycotypes (except the bisecting) was found to be associated with the Ethiopian population. Ethnic-based greater disparities in the serum level of the glycoforms was mainly pronounced towards the higher *m/z* tri-/tetra-sialylated and tri-/tetra-antennary structures. Next to Ethiopians, consistent declining trend in the expression level was noticed in Indians, Japanese, and finally US controls, respectively. Minimum inter-ethnic differences in the level of bisecting glycoforms was observed across all ethnicities.

### 3.4 Sialic acid quantification in human serum

**Fig 8:**
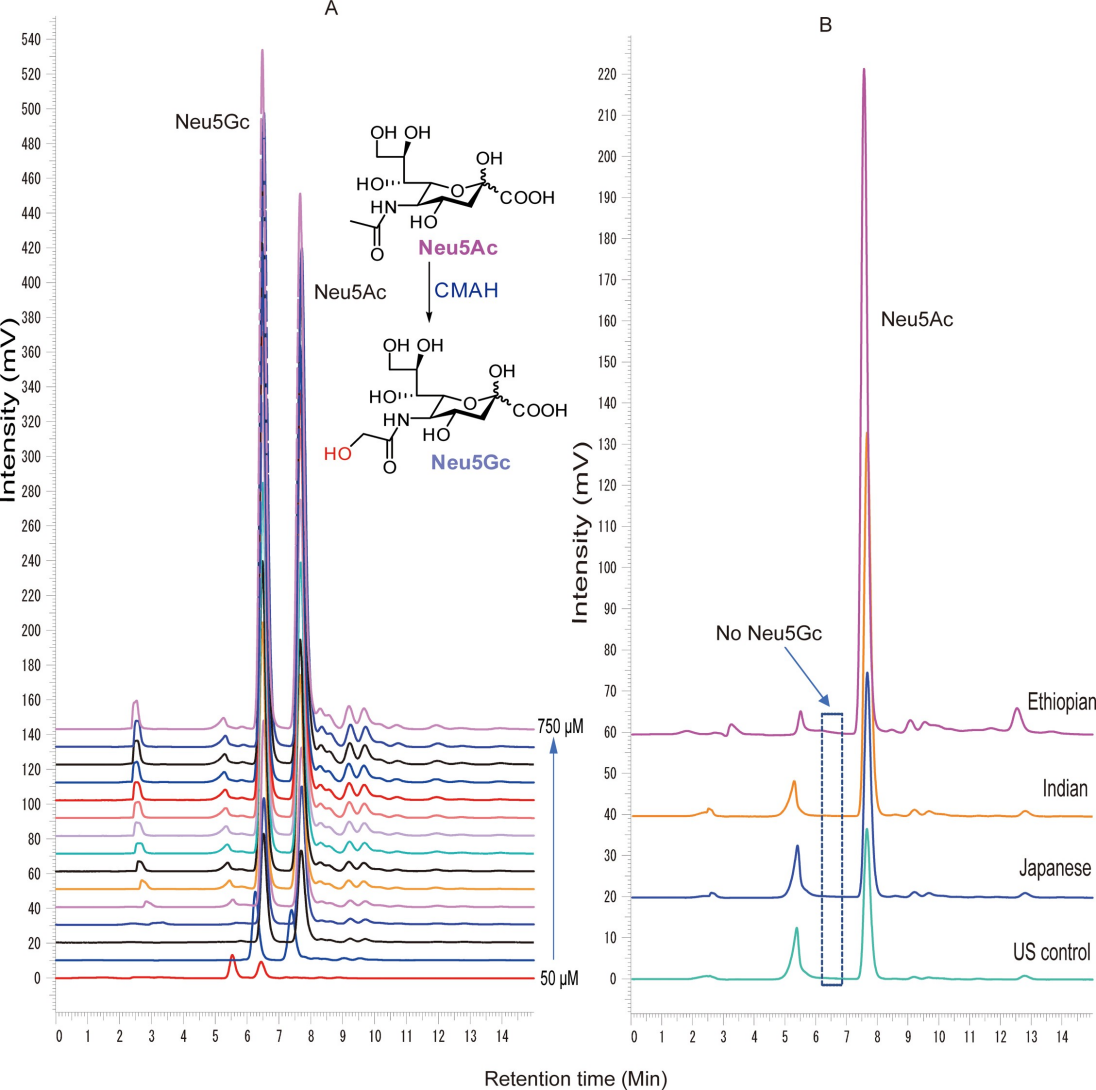
HPLC chromatogram showing quantitation of free sialic acids. **A**) standard Neu5Gc and standard Neu5Ac, **B**) Neu5Ac derived from captured serum *N*-glycans. **Neu5Ac**: *N-*acetylneuraminic acid, **Neu5Gc**: *N-*glycolylneuraminic acid. **CMAH**: Cytidine monophosphate *N*-acetylneuraminic acid hydroxylase (enzyme that catalyzes conversion of Neu5Ac to Neu5Gc).

**Fig 9:**
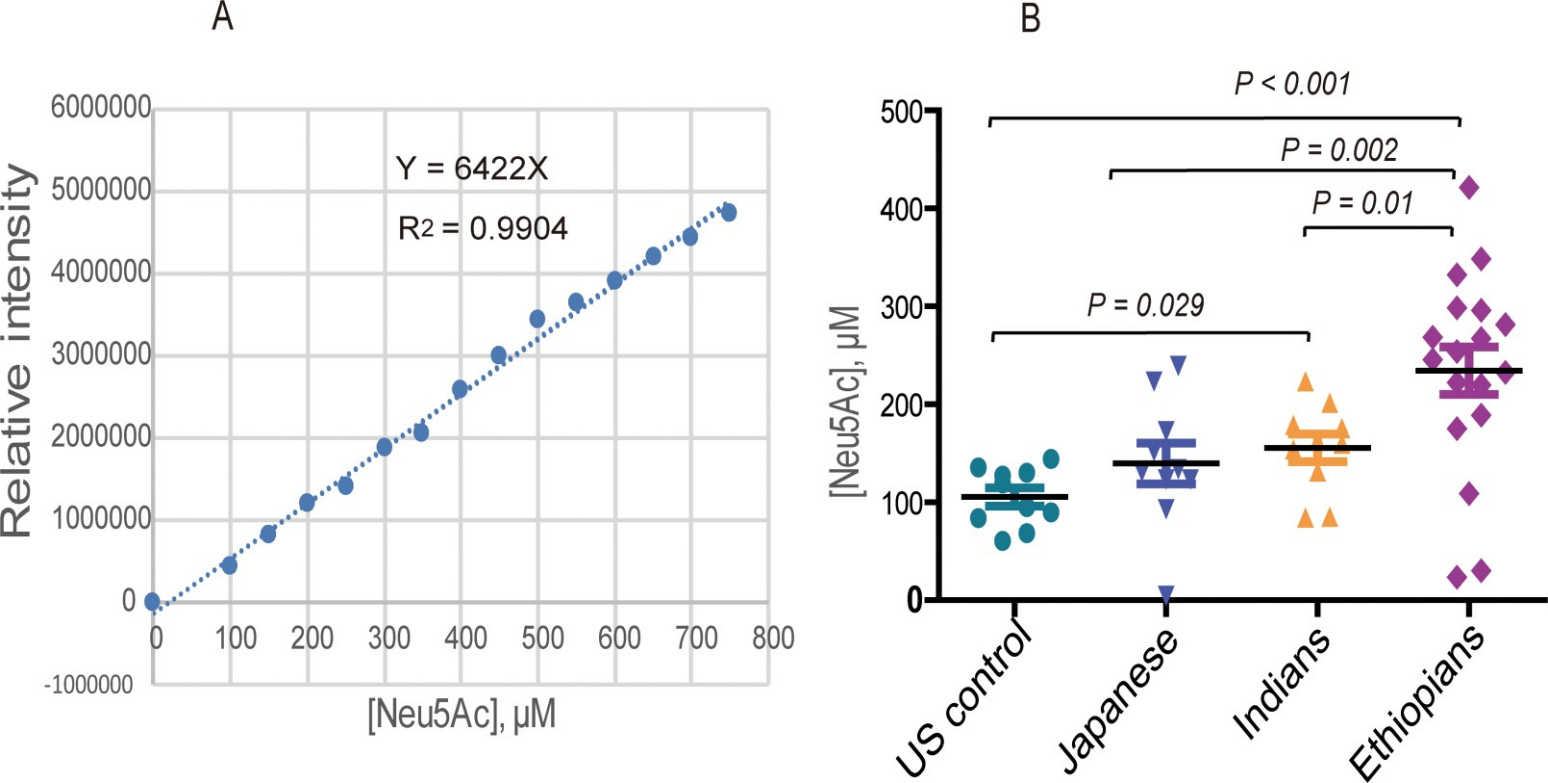
HPLC-based Neu5Ac quantification. **A**) standard Neu5Ac calibration curve, **B**) Dot plot showing Neu5Ac expression levels derived and quantified from human serum *N*-glycans of four ethnic groups.

We also performed a glycoblotting-assisted HPLC-based quantification of free sialic acid species cleaved from sialylated *N*-glycans captured by glycoblotting of human serum glycoproteins. 1,2-diamino-4,5-methylenedioxybenzene (DMB) facilitated selective labeling of sialic acid moieties was performed prior to HPLC-fluorescence detection. Standard solutions of *N*-acetylneuraminic acid (Neu5Ac) and *N*-glycolylneuraminic acid (Neu5Gc) in 50–750 μM concentration ranges were analyzed in parallel with the serum samples. Samples from four ethnic groups (US controls, Japanese, Indians and Ethiopians) were used in this experiment. The result demonstrated a consistent signal intensity pattern for the standard Neu5Gc and Neu5Ac in a concentration dependent manner as shown by HPLC profiles in Fig 8A), suggesting the reliability of the quantification method. Focusing on the study samples, highest peak intensity for Neu5Ac was primarily found in the Ethiopian group (Fig 8B) whose absolute concentration (average μM ± SE = 234.23 ± 24.15), as determined based on the standard Neu5Ac calibration curve (Fig 9A), was significantly higher (*p* < 0.001, *p* = 0.002, *p* = 0.01) comparing with that of US controls (105.51 ± 9.39), Japanese (139.84 ± 20.86), and Indians (155.69 ± 14.27), respectively (Fig 9B). Apart from inter-ethnic variation, Neu5Ac level was observed to be very low in only a few of the Ethiopian subjects, implicating the need to consider inter-individual disparities in the glycosylation process. However, Neu5GC was identified in none of the study subjects which is not surprising as this type of sialic acid normally does not exist in healthy human serum glycoproteins [35]. Furthermore, HPLC chromatogram produced from serum Neu5Ac level of three age-matched females varying in ethnicity (Ethiopian 40 y/o, Indian, 43 y/o, Japanese 40s y/o) is provided in S3 Fig which shows marked differences in their Neu5Ac peak intensity, being most abundant in the Ethiopian subject. This observation was consistent with the result of total serum *N*-glycome spectra of the same individuals [S1 Fig], all of which indicating that healthy human serum *N*-glycosylation pattern seems to be affected more strongly by ethnic difference than gender or age variation of the participants.

## Discussion

Aiming at investigating the inter-ethnic physiological variations in serum *N*-glycome profile, we performed a glycoblotting-assisted MALDI-TOF/MS-based quantitative analysis focusing on 54 healthy subjects of various ethnicity. Total of 51 *N*-glycoforms could be identified and reproducibly quantified with an evident differential expression pattern among the ethnic groups. Among all, Ethiopian ethnic group exhibited the most isolated detection trend with greatly increased expression levels of particularly high mannose, Core-fucosylated, multi-antennary, and multi-sialylated glycans. Notably, some less intense glycans had exclusive occurrence in which 13 glycan structures were specific to Ethiopians whereas other 5 glycoforms were identified to be associated with the remaining ethnicities. Healthy Indian and Japanese serum glycoproteins seem to have mostly similar *N*-glycan composition and abundance with slight differences in the expression level of few *N*-glycoforms. It is interesting to note that one glycoform carrying a core-fucose and a bisecting GlcNAc structures (m/z 2118) showed a clear-cut expression difference among the four study groups. It is the only glycoform whose serum level was markedly decreased in Ethiopians comparing with the other ethnic groups. Declining of this glycan in Ethiopian subjects is most probably due to its involvement as a substrate in the subsequent biosynthetic steps, the product of which (*m/z* 2423 and *m/z* 2728 in Fig 5B) could be up-regulated comparing with the other ethnic populations. We have found high inter-individual variations in the expression level of multiply branched and sialylated glycan structures in the Indian and Japanese subjects (Fig 6). Combining both observations of lower inter-individual variation and higher glycan concentration (compared with Indians and Japanese) found in Ethiopians, it can be suggested that total serum glycan concentration was elevated across the entire Ethiopian subjects. Marked differences in glycotyping analysis, in which up to 2–3 times greater abundance particularly in the multi-branched or multi-sialylated glycan features, were associated with Ethiopian ethnic group (Fig 7). This observation was consistent with the above-mentioned differential expression pattern of the individual *N*-glycans.

Increasing evidences show that glycan abundance is regulated by multiple molecular mechanisms that rely on metabolic interplay among genes, sugars, proteins, and lipids [1, 9]. In glycoproteins, the amino acid sequences of the core protein mostly remain stable while the glycan moiety undergoes faster alterations in response to physiological, pathological, and environmental stimuli [36–38]. Despite previous studies that have reported the correlation between changes in plasma/IgG glycosylation patterns and aging process [26–28], there was no age wise significant variation of healthy serum *N*-glycome profile in the current study, probably due to the relatively younger age range of the healthy participants. With quite different background of our study subjects, the observed changes in the present study may be explained by genetic, environmental, (nutritional habits, physical activity, exposure to pathogens, stress level), and sociocultural variations, while the degree to which each factor influences serum glycan level may depend on the specific ethnic population. These variations have been emphasized by previous reports addressing the variability, heritability, genetic and environmental determinants of human plasma and IgG *N*-glycome using chromatographic techniques [26, 39, 40]. Given the complexity of glycosylation at cellular and molecular levels, these diverse factors can ultimately affect the activity of glycosyltransferases and glycosidases enzymes that orchestrate the glycan biosynthetic and degradative pathways [41]. In this context, despite the non-template-based synthesis of glycans, their structures and expression abundance have been reported to be regulated by genetic and epigenetic factors [42, 43], partly accounting for the inter-individual glycan signature variations observed in health and disease conditions. It was also pointed out that variations in the composition of plasma protein *N*-glycans, particularly increased branching, galactosylation and sialylation features, have been associated with metabolic syndrome related risk factors and higher risk of developing type 2 diabetes [18, 19]. While literatures on the association of healthy human serum glycosylation signatures with ethnicity are scarcely available, numerous studies have witnessed ethnic-specific differences in a number of biochemical markers [44, 45]. Although appreciating population diversity during comprehensive experimental studies is mostly uncommon, we have considered black Ethiopians/Africans in the present study which can be taken as an advantage as pattern of glycosylation in black population, from physiological or pathological perspective, has not been addressed elsewhere.

Surprisingly, some of the specific *N*-glycoforms observed to be exclusive or elevated in the Ethiopian ethnic group including the hyper-branched structures with sialic acid residues (mz 1362, 1591, 3195, 3560, 3865) have previously been identified as sensitive serum biomarkers as a significant recurrence factor of HCC using large-scale Japanese samples [29]. In the present study, these glycans could demonstrate nearly similar serum expression pattern between the Ethiopian group and the HCC group (11 Japanese patients) up on simultaneous experimentation (Fig 5). Similarly, among the hyperbranched glycans strongly increased in Ethiopians (p < 0.001), it was demonstrated that the glycans with m/z 2337, 2439, and 2890 could become promising prognostic biomarkers in renal cell carcinoma [46] while m/z 3049 and 3414 have been associated significantly with metastatic castration-resistant status in prostate cancer [47]. Hence, alteration in serum *N*-glycan profile seems not exclusive for pathological conditions as the present results also clearly demonstrated among healthy subjects in an ethnicity dependent manner. Altogether, these observations emphasize the substantial impact of ethnic differences in human serum *N*-glycome variation, the ignorance of which may provide unclear and imprecise conclusion of the diagnosis by using glycan-related disease biomarkers.

Free sialic acid quantitation result among the four ethnic groups revealed nonnegligible ethnic differences in the serum Neu5Ac level in which the highest abundance has been shown in the Ethiopians, compared to the remaining groups [Figs 8B and 9B]. These informative ethnic-associated variations in the free sialic acid residue further strengthen our MALDI-TOF/MS-based quantification results that demonstrated a consistent declining trend in the expression levels of sialylated glyco-subclasses among Indians, Japanese, and US controls, respectively. The non-detection of Neu5Gc in the current result agrees with the fact that humans do not naturally produce it because of the species-specific embryonic inactivating mutation of the gene encoding for CMP-Neu5Ac hydroxylase enzyme that converts Neu5Ac to Neu5Gc [35]. In addition, it is clear that there is little influence of exogenously incorporated Neu5Gc to the *N*-glycan biosynthesis of major serum glycoproteins. Neu5Ac, being a chief contributor of the anion layer of cellular surfaces in human, greatly modulates cell to cell repulsion, ligand-receptor interaction, immunogenicity, half-life of circulatory proteins, glomerular filtration, neural plasticity and cognitive development [48–50].

Given that more than half of human proteins are glycosylated [51], considerable interest still exists in identifying the specific carrier proteins to which those glycans attach. Bi-antennary serum *N-*glycans are reported to be carried mainly by IgG, a major serum glycoprotein and an essential part of the immune system, whose structural stability, binding and effector functions are greatly influenced by the type of *N*-glycan attached [52]. Recent inter- and intra- population studies on IgG *N*-glycome profile have increasingly evidenced the association between alteration in IgG *N*-glycome profile and hypertension [20–22]. Particularly, ethnic-based differences in IgG *N*-glycome have been observed with significantly reduced galactosylation and sialylation features in European hypertensive subjects (but non-significantly in Chinese cases), comparing to their healthy counterparts [20]. In another IgG subclass-specific *N*-glycomic study, Liu JN et al consistently found a marked decrease in galactosylation of IgG1, IgG2, and IgG4, as well as sialylation of IgG1 and IgG2 among northwestern Chinese hypertensive individuals of four different ethnic categories [22]. Further association of IgG glycosylation alterations (loss of galactose and sialic acid, along with addition of bisecting GlcNAc) with blood lipid profile was proposed to cause dyslipidaemia [24], whereas more core fucose and bisecting GlcNAc structures were found to be strongly associated with atherosclerotic plaque [23]. Apart from the role of IgG glycovariants in switching on and off the pro- and anti-inflammatory functions of IgG (and hence its contribution in disease pathogenesis), these reports are suggestive for the possibility that individual variation in IgG *N*-glycan profile may influence the extent of susceptibility to the conditions of diseases. The hyperbranched and hypersialylated glycans most of which have shown ethnic-based differences in the present study are possibly originated from Alpha-1-acid glycoprotein (AGP). It is one of the heavily *N*-glycosylated serum proteins carrying mainly high molecular weight glycans [53] among of which our group has recently succeeded in developing a focused glycoproteomics strategy to directly quantify serum level of AGP carrying a tri-antennary glycoform in multiple cancer types [54].

The Versatility of our comprehensive glycoblotting method is evident because many glycoforms have reproducibly been profiled from diverse biological samples including serum, cell lines, tissues, and cerebrospinal fluid [11–13, 29–32, 46, 47, 55, 56]. Importantly, unlike several prior reports that had measured relative abundances, our systematic strategy could concurrently quantify the absolute concentrations of whole serum glycome and their free sialic acid terminals only from 10 μL of serum aliquot.

In conclusion, our inter-ethnic group glycomics result strongly revealed noticeable variations among the ethnic populations with high mannose, core-fucosylated, multi-antennary and multi- sialylated glycans, as well as the predominant sialic acid (Neu5Ac) demonstrated highest abundance in Ethiopians. The result further indicated some of the glycans that have shown profound expression alteration may not be useful candidates to be biomarkers of various diseases due to their large inter-ethnic and inter-individual variation. Despite the general scope of the present study, we were able to obtain interesting and informative results on the associations between ethnic difference and distinct changes in protein glycosylation which may become helpful for further in-depth investigations in the area. Due to limited samples to comprehensively address the gender and age effects, the current results are preliminary, and thus cannot be generalized to the target populations. In a large-scale study employing these and other ethnic compositions, we further need to investigate the correlation between human glycoforms and various confounding factors including gender and age. Establishing database for healthy human glycome variations among multi-ethnic populations is important as it further improves and accelerates the clinical utility of glycomics and glycoproteomics fields.

## Supporting information

S1 Fig Comparative serum *N*-glycome spectra of age-matched females varying in ethnicity. In attempt to provide more clarity on whether difference in ethnicity or gender had marked effect on the *N*-glycan profile, we have included one Indian female (age = 43) and one Japanese female (age = 40s), whose serum *N*-glycome spectra is comparatively presented with that of one Ethiopian female (age = 40). Some of the *N*-glycan peaks showing marked variations among the three female subjects are highlighted by the dotted line shape. Considering this result from gender and age matched samples, most serum glycoforms showed abundant peak intensity in the Ethiopian subject, while one triantennary trisialylated glycan (m/z ^˷^3049) demonstrated highest intensity in the Indian sample. These results from few female subjects intensify the variations observed in serum *N*-glycan profile result when Indian, Japanese, and US male subjects were considered as well, evidencing the profound influence of ethnicity on the *N*-glycosylation signature of the study groups.
(TIF)

S2 Fig Serum *N*-glycome spectra showing Indian and Japanese of both sexes. Age of subjects: Indian female = 43, Indian male = 39, Japanese female = 40s, Japanese male = 30s. Variation in *N*-glycan peak intensity is clearly shown in an ethnicity dependent manner in which glycans such as the core-fucosylated (m/z 1591 and 1753) illustrated abundant expression in the Japanese subjects, whereas hyperbranched and hypersialylated glycans (m/z 3049 and 3195) demonstrated up-regulated expression in the Indian subjects, irrespective of the gender difference.
(TIF)

S3 Fig HPLC chromatogram of free Neu5Ac captured from serum of three age-matched females varying in ethnicity. The female subjects were each of Ethiopian (age = 40), Indian (age = 43), and Japanese (age = 40s). This result from age- and gender-matched subjects provides an evidence for the strong influence of ethnic deference on the sialylation pattern of human serum glycoproteins and strengthens our total sialylated *N*-glycan result that demonstrated ethnic-associated variation in detection profile.
(TIF)

S1 Table Demographic characteristics of study participants. While recruiting the Japanese subjects, age of each subject was not recorded in its exact value, rather as 20s (within 20–29.9 y/o), 30s (within 30–39.9 y/o), 50s (within 50–59.9 y/o), 60s (within 60–69.9 y/o). The US origin control serum is a pool collected from several male donors whose age information was not provided as it was purchased sample from Sigma-Aldrich company, product # H4522.
(DOCX)

S2 Table List of N-glycans considered for glyco-subclass analysis. Only glycans that were detected in all the ethnic groups have been considered for the glyco-subclass analysis. There is a chance that a glycan can be counted in more than one group when it contains more than one structural features as per the grouping mechanism. m/z values are given as label with each glycan structure.
(DOCX)
