# Supplementary figures and images for: Healthy human serum N-glycan profiling reveals the influence of ethnic variation on the identified cancer-relevant glycan biomarkers
Source: PLoS One. 2018 Dec 28;13(12):e0209515. doi: 10.1371/journal.pone.0209515 (PMC6310272; doi:10.1371/journal.pone.0209515)

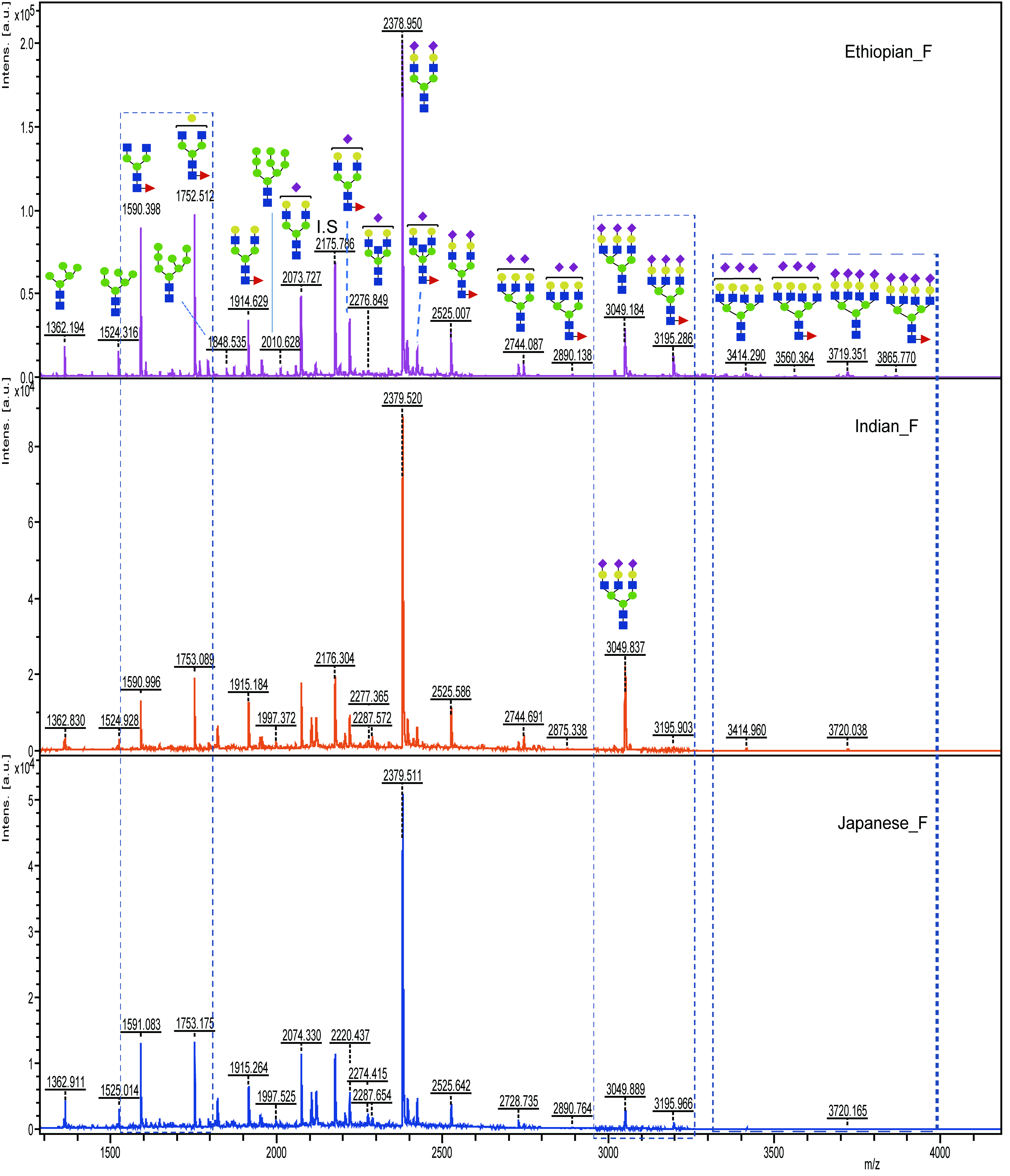

Supplement: S1 Fig — In attempt to provide more clarity on whether difference in ethnicity or gender had marked effect on the N-glycan profile, we have included one Indian female (age = 43) and one Japanese female (age = 40s), whose serum N-glycome spectra is comparatively presented with that of one Ethiopian female (age = 40). Some of the N-glycan peaks showing marked variations among the three female subjects are highlighted by the dotted line shape. Considering this result from gender and age matched samples, most serum glycoforms showed abundant peak intensity in the Ethiopian subject, while one triantennary trisialylated glycan (m/z ˷3049) demonstrated highest intensity in the Indian sample. These results from few female subjects intensify the variations observed in serum N-glycan profile result when Indian, Japanese, and US male subjects were considered as well, evidencing the profound influence of ethnicity on the N-glycosylation signature of the study groups. (TIF) [file pone.0209515.s001.tif]

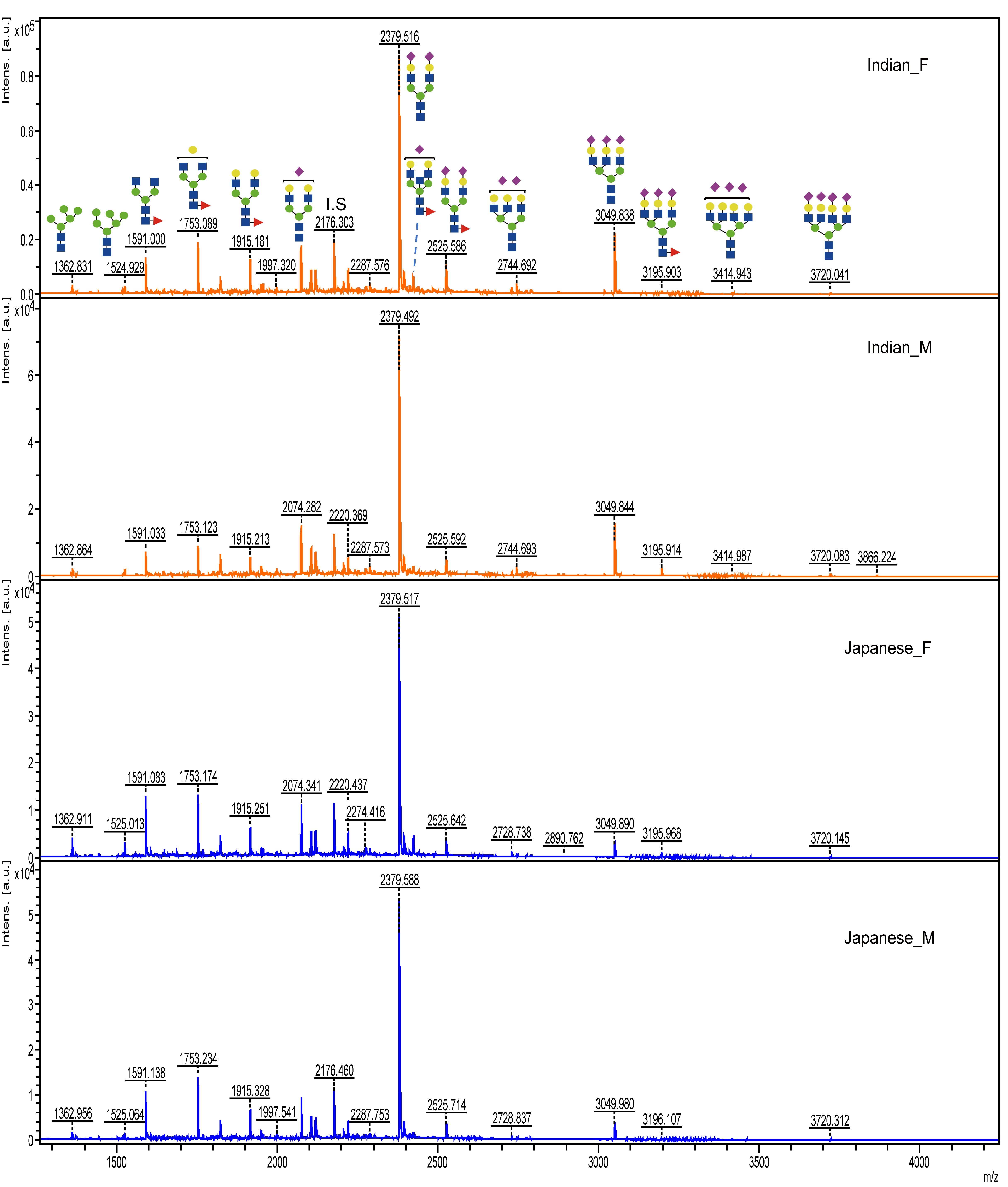

Supplement: S2 Fig — Age of subjects: Indian female = 43, Indian male = 39, Japanese female = 40s, Japanese male = 30s. Variation in N-glycan peak intensity is clearly shown in an ethnicity dependent manner in which glycans such as the core-fucosylated (m/z 1591 and 1753) illustrated abundant expression in the Japanese subjects, whereas hyperbranched and hypersialylated glycans (m/z 3049 and 3195) demonstrated up-regulated expression in the Indian subjects, irrespective of the gender difference. (TIF) [file pone.0209515.s002.tif]

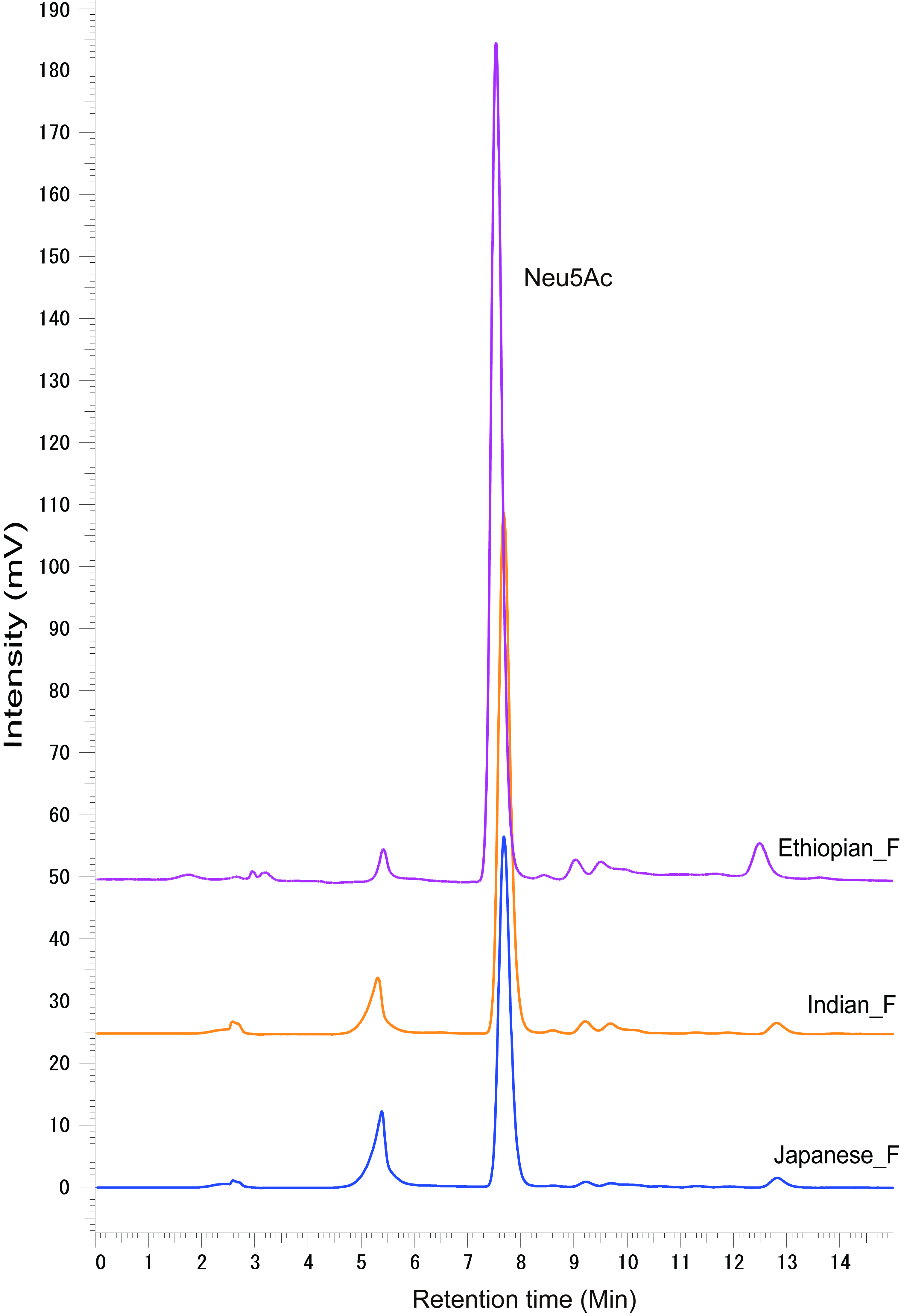

Supplement: S3 Fig — The female subjects were each of Ethiopian (age = 40), Indian (age = 43), and Japanese (age = 40s). This result from age- and gender-matched subjects provides an evidence for the strong influence of ethnic deference on the sialylation pattern of human serum glycoproteins and strengthens our total sialylated N-glycan result that demonstrated ethnic-associated variation in detection profile. (TIF) [file pone.0209515.s003.tif]

| **Core-fuco** | **Bisecting** | **Mono/Di-Sia** | **Tri/Tetra-Sia** | **Tri/Tetra-Anten** |
| --- | --- | --- | --- | --- |
| 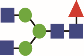 1591 | 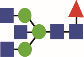1794 | 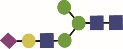1709 | 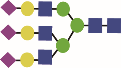3049 | 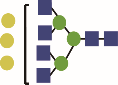2337 |
| 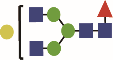1753 | 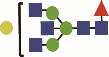1956 | 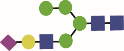1871 | 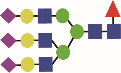3195 | 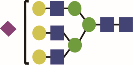2439 |
| 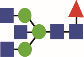1794 | 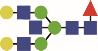2118 | 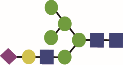2033 | 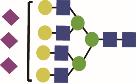3414 | 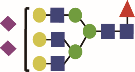2890 |
| 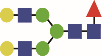1915 | 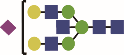2277 | 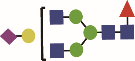2058 | 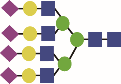3719 | 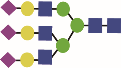3049 |
| 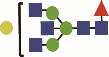1956 | 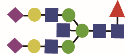2728 | 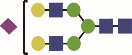2074 |  | 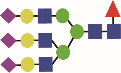3195 |
| 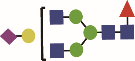2058 |  | 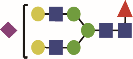2220 |  | 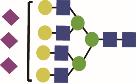3414 |
| 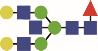2118 |  | 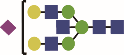2277 |  | 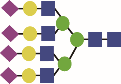3719 |
| 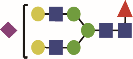2220 |  | 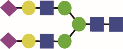2379 |  |  |
| 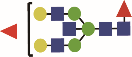2264 |  | 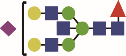2423 |  |  |
| 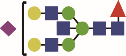2423 |  | 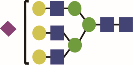2439 |  |  |
| 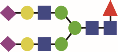2525 |  | 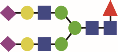2525 |  |  |
| 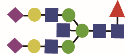2728 |  | 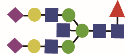2728 |  |  |
| 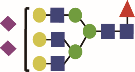2890 |  | 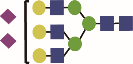2744 |  |  |
| 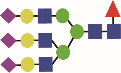3195 |  | 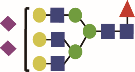2890 |  |  |

Supplement: S2 Table — Only glycans that were detected in all the ethnic groups have been considered for the glyco-subclass analysis. There is a chance that a glycan can be counted in more than one group when it contains more than one structural features as per the grouping mechanism. m/z values are given as label with each glycan structure. (DOCX) [file pone.0209515.s005.docx]
