# Supplementary material for: Healthy human serum N-glycan profiling reveals the influence of ethnic variation on the identified cancer-relevant glycan biomarkers
Source: PLoS One. 2018 Dec 28;13(12):e0209515. doi: 10.1371/journal.pone.0209515 (PMC6310272; doi:10.1371/journal.pone.0209515)
Supplement: S1 Table — While recruiting the Japanese subjects, age of each subject was not recorded in its exact value, rather as 20s (within 20–29.9 y/o), 30s (within 30–39.9 y/o), 50s (within 50–59.9 y/o), 60s (within 60–69.9 y/o). The US origin control serum is a pool collected from several male donors whose age information was not provided as it was purchased sample from Sigma-Aldrich company, product # H4522. (DOCX) [file pone.0209515.s004.docx]

|  |  |  |  |  | *Age (years)* | |
| --- | --- | --- | --- | --- | --- | --- |
| *Category* |  | *Number (n)* | *Gender* |  | *Mean ± SD* | *Range* |
| US control | 10 | | M | --- | | --- |
| Japanese | 10 | | M |  | | 20s-30s |
| Indian | 10 | | M | 32.5 ± 5.039 | | 27-43 |
| Ethiopian | 24 | | F | 31.54 ± 7.175 | | 23-50 |
| HCC | 11 | | M | --- | | 50s-60s |
| Total | 65 | |  |  | |  |
